# Study on the Traffic Air Pollution inside and outside a Road Tunnel in Shanghai, China

**DOI:** 10.1371/journal.pone.0112195

**Published:** 2014-11-11

**Authors:** Rui Zhou, Shanshan Wang, Chanzhen Shi, Wenxin Wang, Heng Zhao, Rui Liu, Limin Chen, Bin Zhou

**Affiliations:** 1 Shanghai Key Laboratory of Atmospheric Particle Pollution and Prevention (LAP3), Department of Environmental Science & Engineering, Fudan University, Shanghai 200433, China; 2 School of Environment and Architecture, University of Shanghai for Science and Technology, Shanghai 200093, China; 3 Fudan Tyndall Centre, Fudan University, Shanghai 200433, China; Tsinghua University, China

## Abstract

To investigate the vehicle induced air pollution situations both inside and outside the tunnel, the field measurement of the pollutants concentrations and its diurnal variations was performed inside and outside the Xiangyin tunnel in Shanghai from 13:00 on April 24th to 13:00 on April 25th, 2013. The highest hourly average concentrations of pollutants were quantified that CO, NO, NO_2_ and NO_X_ inside the tunnel were 13.223 mg/m^3^, 1.829 mg/m^3^, 0.291 mg/m^3^ and 3.029 mg/m^3^, respectively, while the lowest ones were 3.086 mg/m^3^, 0.344 mg/m^3^, 0.080 mg/m^3^ and 0.619 mg/m^3^. Moreover, the concentrations of pollutants were higher during the daytime, and lower at night, which is relevant to the traffic conditions inside the tunnel. Pollutants concentrations inside the tunnel were much higher than those outside the tunnel. Then in a case of slow wind, the effect of wind is much smaller than the impact of pollution sources. Additionally, the PM_2.5_ concentrations climbed to the peak sharply (468.45 µg/m^3^) during the morning rush hours. The concentrations of organic carbon (OC) and elemental carbon (EC) in PM_2.5_ inside the tunnel were 37.09–99.06 µg/m^3^ and 22.69–137.99 µg/m^3^, respectively. Besides, the OC/EC ratio ranged from 0.72 to 2.19 with an average value of 1.34. Compared with the results of other tunnel experiments in Guangzhou and Shenzhen, China, it could be inferred that the proportion of HDVs through the Xiangyin tunnel is relatively lower.

## Introduction

The primary air pollutants emitted by motor vehicles are carbon monoxide (CO), nitrogen oxides (NO_X_, including NO and NO_2_), hydrocarbons (HCs) and particulate matter (PM). Vehicular exhaust has become a main source of air pollution. Among these emissions, CO and NO_X_ emissions are accounting for more than 80% and 40% of the total urban emissions in many big cities like Beijing, Shanghai and Guangzhou in China [1]–[3]. The particles from vehicular exhaust are mainly composed of organic carbon (OC) and elemental carbon (EC); and the combinational effect of OC and EC can reduce visibility, accounting for 30% to 40% of the total extinction [4]. Moreover, the HC and NO_X_ are precursors to secondary ozone formation and aerosols. These products and the vehicle-induced pollutants can cause damages to human health, including the harms to respiratory system, cardiovascular system etc. [5], [6].

With the increasing recognition of the adverse effects of vehicle-induced air pollution and the relationship between the pollutants and diseases, some solutions have been considered [7]. For example, constructing tunnels to redirect traffic away from surface roads not only alleviates the increasing serious problem of traffic congestion, but also largely improves ambient air quality [8]. However, it has also been found that inadequate ventilation in the tunnels combined with high traffic volume can result in elevated concentrations of vehicle-induced air pollutants. He [9] characterized comprehensively the PM_2.5_ emissions inside the Zhujiang Tunnel in the Pearl River Delta region of China. It was found that the organic compounds in vehicular PM_2.5_ emissions were less influenced by the geographic area and fleet composition. Thereby it is more suitable for across extensive regions to use in aerosol source apportionment modeling. The diffusion of exhaust gas jetted out from city traffic tunnel under different conditions of the structure size of wind tower, jet velocity of exhaust gas and relevant ambient meteorological parameters, and the decay rates of exhaust gas were discussed by Tian [10]. Compared with the environmental effect of shaft discharge with cavern mouth diffusion by the Gauss model and TOP model theory, it was suggested that the environmental pollution contribution of CO is remarkably weaker than that of NO_2_ under the same conditions. Besides, the environmental effect of cavern mouth diffusion, which is affected by air direction evidently, is more serious than that of shaft discharge [11].

The aim of this study is to figure out the characteristics of the concentrations of vehicle-induced air pollutants inside and outside the tunnel (the Xiangyin tunnel, Shanghai) over a 24-hours period, as well as the relationship between these variables. Furthermore, the carbonaceous substances from PM_2.5_ in the tunnel, namely OC and EC, along with their ratio were discussed.

## Methodology

### Experimental location

The Xiangyin tunnel is located in the northeast of Shanghai, which is crossing the Huangpu River. It is an urban two-bore tunnel (north and south bores) with two lanes of traffic per bore (without walkways). The tunnel has a length of 2.6 km with designed speed capacity of 80 km/h, and the ventilation mode is longitudinal. Two exhausting towers are located on the east side of the Huangpu River (about 120 m away from the exit), and another same set on the west side, while two horizontal axial exhausting fans are set to centralize and diffuse exhausts. The volume flow rate, power and pressure of the fans are 125 m^3^/s, 250 kW and 1000 Pa, respectively. There were no specific permissions required for this experiment location, and the experiment study did not involve endangered or protected species.

### Monitoring sites and instruments

The monitoring sites (121.58°E,31.30°N) were positioned on the east side of the exhausting tower, both inside and outside the tunnel. The monitoring time was from 13:00 on April 24^th^ to 13:00 on April 25^th^, 2013, during which one horizontal axial exhausting fan was working from 7:00 to 10:00 on April 25^th^. The monitoring site outside the tunnel was to the west of the exhausting tower, which was located on the east side of the Huangpu River. And the online data about meteorology and pollutants outside the tunnel were gained with a monitoring van at the monitoring site. The meteorological data included temperature, relative humidity, atmospheric pressure, as well as wind speed and direction. The pollutants were mainly carbon monoxide (CO), nitrogen oxides (NO_X_), nitric oxide (NO) and nitrogen dioxide (NO_2_).

The concentration of CO was measured by a carbon monoxide analyzer (Model 48i, Thermo scientific), which used gas filter correlation technology to detect the amount of CO. The concentration of NO-NO_2_-NO_X_ mixture was measured by a nitrogen oxides analyzer (Model 42i, Thermo scientific) using chemiluminescence.

Inside the tunnel, the monitoring site was located at the top of south bore of the tunnel, which was 10 m away from the exhausting fan. The monitoring instruments used inside the tunnel were same as those used outside the tunnel to measure the concentrations of the same pollutants (CO, NO, NO_2_, NO_X_).

### Sampling and instruments

For the PM_2.5_ samples, the sampling site was located at the top of south bore of the tunnel, the same as the monitoring site inside the tunnel. The sampling time was from 13:00 on April 24^th^ to 11:00 on April 25^th^ with the temporal resolution of an hour. PM_2.5_ concentrations were measured by the gravimetric method, while the glass fiber membrane filters and an intelligent medium volume PM_2.5_ sampler (Model 2030, Laoying, Qingdao Laoshan Applied Technology Institute) were used. The flow rate of the sampler was set at 100 L/min, and the cumulative sample volume was saved automatically after each sampling. In order to determine the PM_2.5_ mass concentrations, glass fiber membrane filters were pro- and post- weighted at least twice on an analytical balance, with a sensitivity of 0.1 mg. Before weighing, the membrane filters were equilibrated in a temperature humidity chamber at 20°C with a relative humidity of 50±5% for 24 hours.

To determine the mass concentrations of OC and EC, the samples were analyzed by a Thermal/Optical Carbon Analyzer (Model 2001A, Desert Research Institute). The technical method of the analyzer is thermo-optical reflection. The operation of the analyzer is based on the preferential oxidation of OC compounds and elemental EC at different temperatures. It relies on the fact that organic compounds can be volatilized from the sample deposit in a non-oxidizing helium (He) atmosphere, while elemental carbon must be combusted by an oxidizer. In this study only 7 samples (April 24^th^ 16:00, 19:00, 22:00; April 25^th^ 1:00, 4:00, 7:00 and 10:00) were actually analyzed.

### Statistical analysis methods

Correlation analysis was used to illustrate the correlation of two variables, which is performed by OriginPro 8.1 software (OriginLab Cooperation). To test the significance of the relationship between variables, the hypothesis test was taken for the correlation coefficient. P-values [12], [13] are often coupled to a significance or alpha (α) level, which is also set ahead of time, usually at 0.05 (5%). Other significance levels, such as 0.1 or 0.01, are also used, depending on the field of study; P-value was set as 0.01 in this study. Thus, if a P-value was found to be less than 0.01, then the result would be considered statistically significant and then the null hypothesis would be rejected. By Origin 8.1 software, correlation coefficient (r) was calculated, and based on the degree of freedom (ν), the corresponding r_α_ could be taken from the correlation coefficient r boundary value table (Table S1). If r≧r_α_, then P<0.01, which means the correlation between the variables significant.

## Results and Discussion

### Traffic volume and pollutants concentrations inside the tunnel

**Figure 1:**
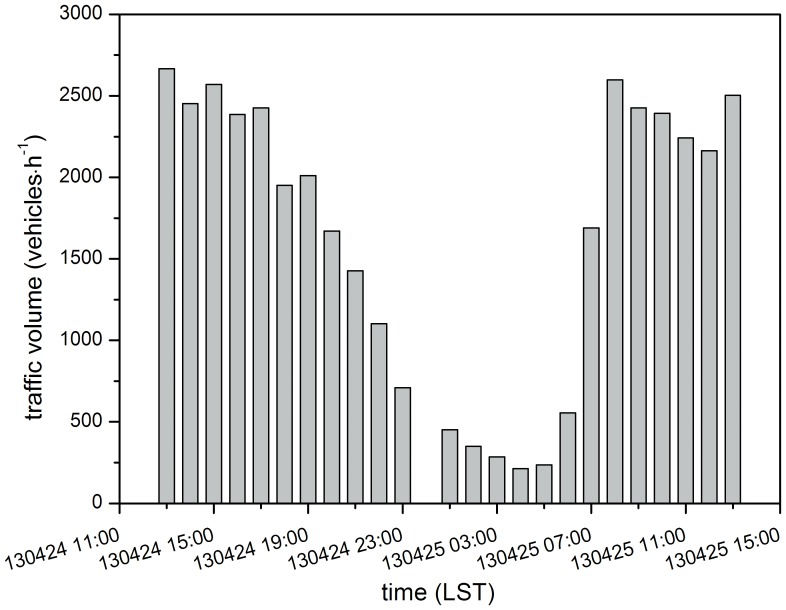
Traffic volume from 13:00 on April 24^th^ to 13:00 on April 25^th^ in the Xiangyin tunnel (south bore).

Fig. 1 displays the traffic volume from west to east direction, i.e. south bore of the tunnel, during the monitoring period (the data for 0:00–1:00 on April 25^th^ was missing). In the daytime (8:00–20:00), the traffic volume were around 2000 vehicles per hour and the maximum occurred between 13:00 and 14:00 on 24^th^ with 2666 vehicles passed through the tunnel. In contrast, the traffic volume declined during the night, with the lowest volume only of 214 vehicles during the 4:00–5:00 period. Due to the heavy congestion and consequent slow movement of traffic, in the morning rush hour the actual number of cars passing through the tunnel was not that large in spite of how busy the road appeared. However, the road appeared relatively quiet in the afternoon, as a similar number of vehicles passed through the tunnel quickly. Therefore, the hourly traffic volume did not differ greatly during the daytime.

**Figure 2:**
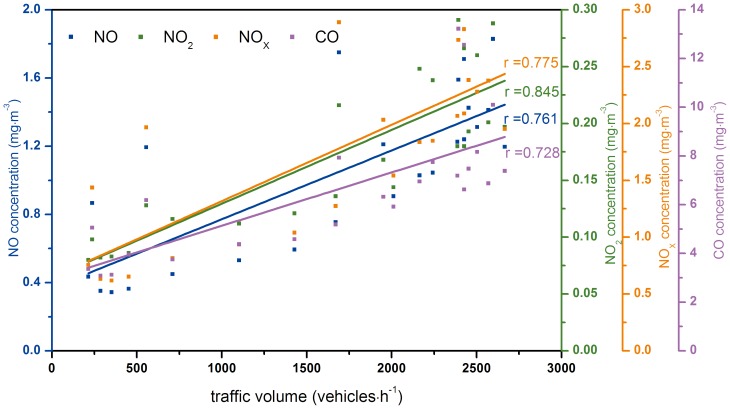
The correlation between the traffic volume and pollutants (NO, NO_2_, NO_X_, CO) concentrations inside the tunnel.

Fig. 2 shows the correlations between the traffic volume and the concentrations of pollutants (NO, NO_2_, NO_X_, CO) inside the tunnel. The correlation coefficients (r) of the traffic volume and the concentrations of NO, NO_2_, NO_X_, CO were 0.761, 0.845, 0.775 and 0.728 respectively, from which it could be assumed that the concentrations of the pollutants were all associated with the traffic volume. Moreover, according to the statistics results, the degree of freedom (ν) was equal to 22 and the corresponding r_α_ was 0.515 referred to the correlation coefficient r boundary value table. It was obvious that the correlation coefficients were all greater than r_α_. Consequently, P-Values were all smaller than 0.001 suggesting the relationships between the traffic volume and pollutant concentrations were significant. However, the traffic volume was not the only factor that determines the pollutants concentrations. Those concentrations were determined by many factors, such as vehicle speed, vehicle type, and fuel, etc. [14]. Due to the limitation of this experimental design, only the traffic volume was monitored and the other information about the traffic condition was unknown.

### Comparison of pollutants concentrations inside and outside the tunnel

**Figure 3:**
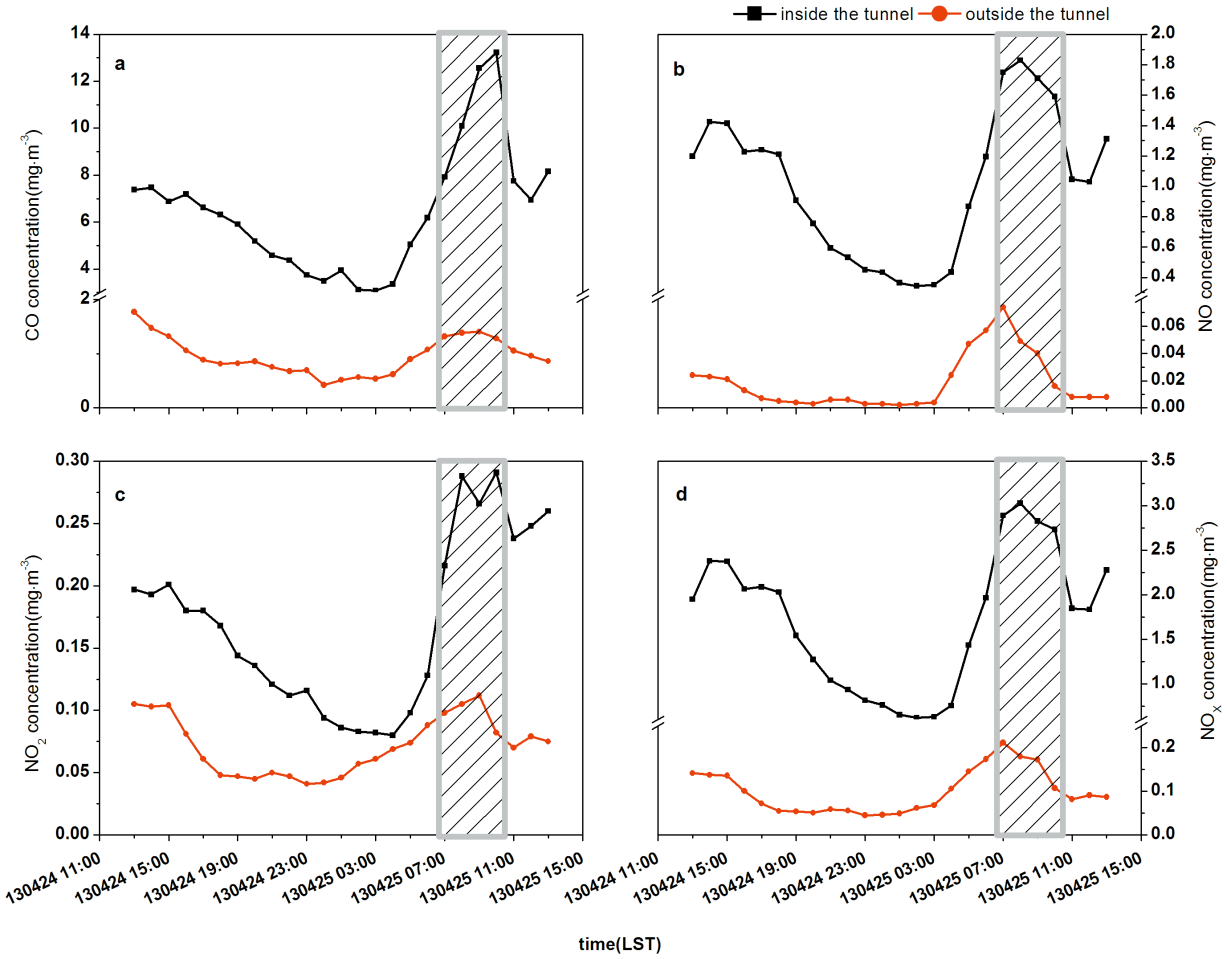
Comparisons of pollutants concentrations inside and outside the tunnel (a: CO, b: NO, c: NO_2_, d: NO_X_; shaded areas: the period of the fan working).

Comparisons of the hourly average concentrations of each pollutant inside and outside the tunnel, obtained using the real-time monitoring instruments, are shown in Fig. 3.

The variations of hourly averaged CO concentrations inside and outside the tunnel were illustrated in Fig. 3.a. Higher concentrations appeared in the morning rush hours, while the values declined to the lowest at midnight. However, the concentrations of CO rose quickly to a peak level during the 8:00–10:00 period. This was associated with traffic congestion so the fact that a large amount of CO was accumulated without diffusion in time. The exhausting fan was working at this time so a portion of CO that accumulated in the tunnel was discharged, which would made the CO concentration of the monitoring site (at the top of the tunnel) increase. Besides, CO concentrations inside the tunnel were much higher than those outside the tunnel by 6 to 7 times on average. The main reasons were that the traffic volume on the surface road was less than that inside the tunnel. Moreover, compared to the relatively isolated environment in the tunnel, the surface road was in an open environment, which was more conducive to the diffusion of CO.

Comparing the NO concentrations inside the tunnel with those outside the tunnel in Fig. 3.b, it could be concluded that concentrations were higher in the daytime (especially in the morning) and lower at night. Furthermore, NO concentrations inside the tunnel were almost 100 times higher than that outside the tunnel. The trend of NO concentrations in the tunnel was mainly associated with the traffic situation and the effect of the fan since NO increased sharply when the fan was working. The situation was more complicated outside the tunnel since there were some important chemical reactions involved. NO could react quickly with O_3_ to form NO_2_, but NO_2_ could also be decomposed back to NO in the condition of the light. Due to these chemical reactions, NO concentrations outside the tunnel were quite low at night, and then rose in the daytime.

In Fig. 3.c, the trends of NO_2_ concentrations inside and outside the tunnel were not similar. The concentrations of NO_2_ were much lower than the other pollutants inside the tunnel. However, the trend of NO_2_ concentrations was similar to those of CO and NO, for which the concentrations in the daytime were higher than at night, and the highest concentrations occurred during rush hours. The variation of NO_2_ concentrations outside the tunnel was not significant, with a maximum value that was only twice the minimum, whereas the ratios of the maximum and minimum values of CO and NO were 4.15 and 37, respectively. NO_2_ was not the main pollutant emitted directly by vehicles. In addition, the concentration of ozone was low inside the tunnel in the general situation, which means that the amount of NO_2_ generated from the reaction with NO was low. Outside the tunnel, NO_2_ could be generated quickly from NO, and with the presence of the light, NO_2_ could revert back to NO. Therefore, the concentrations of NO_2_ didn't change too much overall.

**Figure 4:**
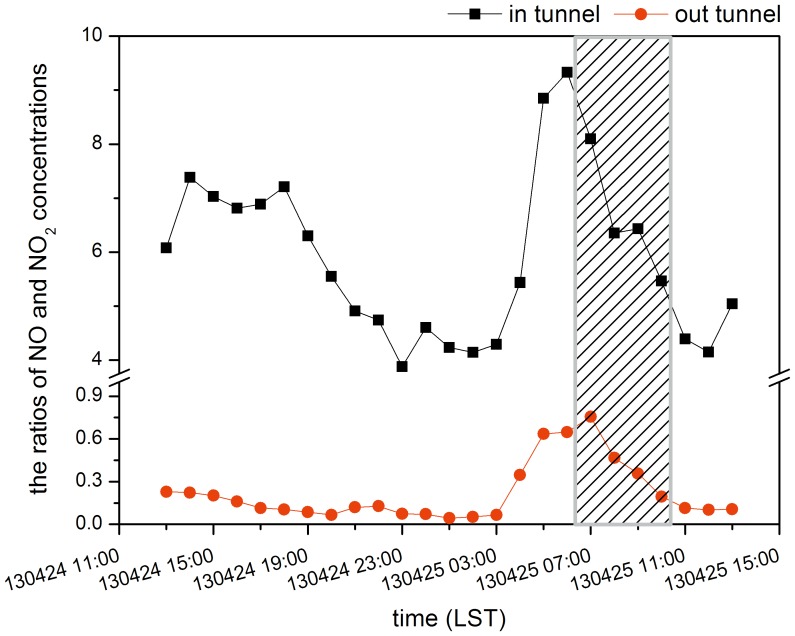
Ratios of NO and NO_2_ concentrations inside and outside the tunnel (shaded area: the period of the fan working).

**Figure 5:**
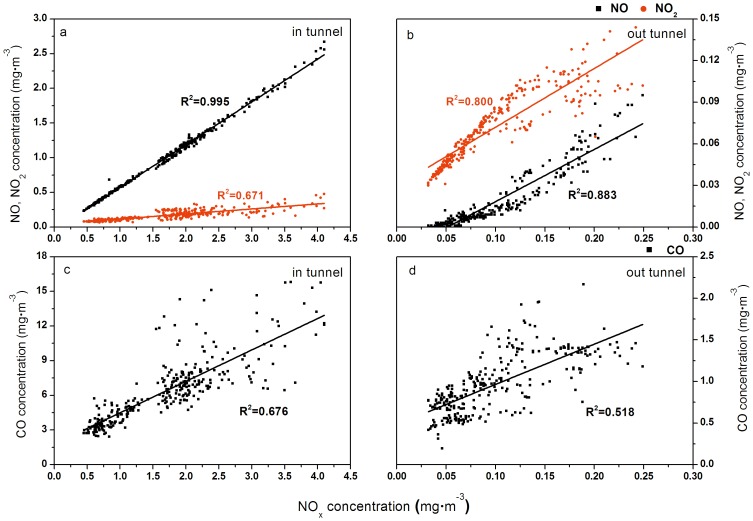
Fitting results of NO, NO_2_, CO with NO_X_ (a: NO, NO_2_ inside the tunnel, b: NO, NO_2_ outside the tunnel, c: CO inside the tunnel, d: CO outside the tunnel).

As the main components concentrations of NO_X_, NO and NO_2_, their ratios inside and outside the tunnel were shown in Fig. 4. The concentration of NO in the tunnel was much higher than NO_2_ (about 6 times), implying NO_X_ in the tunnel was primarily composed of NO. But the situation outside the tunnel was almost the opposite. NO_2_ was much higher than NO except for the early morning of April 25^th^ when the concentration of NO climbed slightly to around two thirds of that of NO_2_. The variation in NO_X_ concentrations can be found from Fig. 3.d and Fig. 5.a and b, which shows the results of fitting linear models to the NO, NO_2_ and NO_X_ data for the inside and outside the tunnel, respectively. Fig. 5.a shows that the correlation coefficient (r) of NO and NO_X_ concentrations was 0.998, while that of NO_2_ and NO_X_ concentrations was 0.820. Inside the tunnel the stronger correlation between NO_X_ and NO implies that NO was a more important component of NO_X_ than NO_2_. As mentioned above, the NO concentration was much higher than NO_2_. As a result NO_X_ concentrations were mainly determined by NO concentrations. Compared with the results inside the tunnel, there was little difference between the correlation coefficient (r) of NO and NO_X_ concentrations and that of NO_2_ and NO_X_ concentrations outside the tunnel from Fig. 5.b. They were 0.895 and 0.940, respectively, which means NO_X_ concentrations outside the tunnel depended on both NO and NO_2_. Thus, the trend of NO_X_ concentrations inside the tunnel was close to the trend of NO concentrations, while outside the tunnel the trend of NO_X_ concentrations was between the trend lines of NO and NO_2_ concentrations and relatively closer to that of NO (Fig. 3). Besides, comparing the correlation coefficients of NO_X_ and CO concentrations (Fig. 5.c and d), we could further analyze the sources of NO_X_ inside and outside the tunnel. The correlation coefficients were 0.823 and 0.721 inside and outside the tunnel, respectively, and showing the NO_X_ inside the tunnel comes from the same source as CO, i.e. traffic exhaust. On the other hand, the sources of NO_X_ outside the tunnel included a small contribution from nearby industrial sources and the traffic exhaust.

### Meteorological conditions and pollutant concentrations outside the tunnel

**Figure 6:**
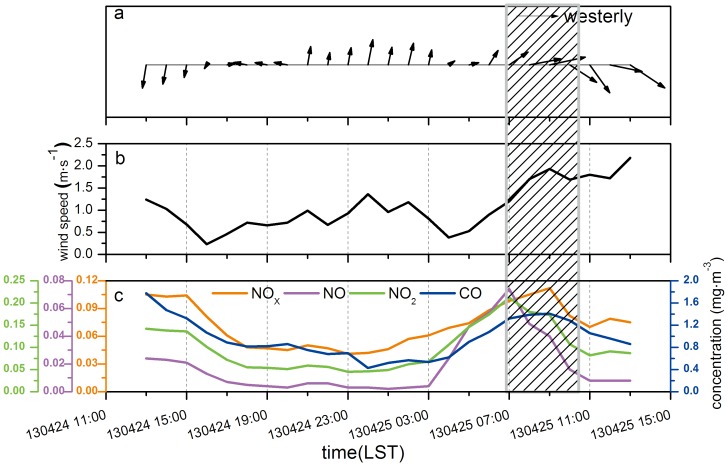
Hourly average meteorological parameters and pollutants concentrations measured by the monitoring van outside the tunnel (a: wind direction, b: wind speed, c: pollutants concentrations; shaded area: the period of the fan working).

By analyzing the variation of the pollutants concentrations inside and outside the tunnel, the sources of pollution and some chemical reactions had a major influence on the pollutant concentrations outside the tunnel. Fig. 6 shows the meteorological parameters measured by the monitoring van during the monitoring period, including the hourly average wind speed and direction. It seems the influence of meteorological conditions was little.

According to Fig. 6.a, at the height of 7 m, the main wind direction was northeast in the afternoon of 24^th^, and then it changed to southeast from the evening of 24^th^ until the early morning of 25^th^. The prevailing wind directions were southwest and northwest during the morning. The wind speed changed a little as shown in Fig. 6.b, from about 1 m/s since the afternoon of 24^th^ till the early morning of 25^th^ to about 1.5–2.5 m/s during the morning of 25^th^. Compared with the trends in pollutant concentrations in Fig. 6.c, the wind direction and speed had hardly any effect on the diurnal variation of pollutant concentrations. Diurnal variations of pollutants concentrations were mainly determined by the sources of pollution and some chemical reactions. For most of the monitoring period, the fan was not working; the sources of pollution were the traffic and industry nearby. However, when the fan was working during the morning rush hours (7:00–10:00 on 25^th^), the pollutants emitted through the wind tower, which might have been affected by ambient wind, could influence the pollutants concentrations measured by the monitoring van. Then this time period was chosen to analyze whether wind direction and speed affect pollutant concentrations.

**Figure 7:**
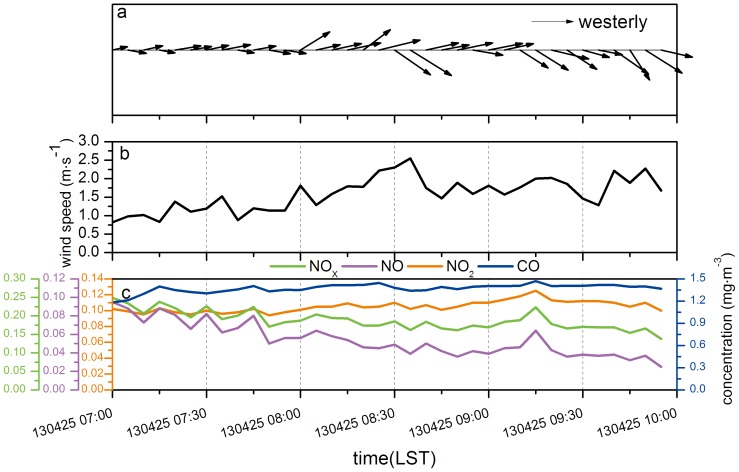
Average meteorological parameters and pollutants concentrations in 5 minutes intervals from 7:00 to 10:00 on April 25^th^ (a: wind direction, b: wind speed, c: pollutants concentrations).

Fig. 7 shows the average meteorological parameters and pollutants concentrations over 5 minutes intervals from 7:00 to 10:00 on April 25^th^. During these three hours, the dominant direction was westerly from Fig. 7.a. During the first one and a half hours the wind direction was mostly WSW, then it turned to WSW from WNW between 8:30 and 9:00. The prevailing wind direction was northwest in the last one hour. Also the wind speed of the northwest wind was greater than that of the southwest wind. Comparing with the trends of NO, NO_2_ and NO_X_ concentrations, when the wind speed was low, the concentration was high. As a result, the speed of the wind might affect the pollutants concentrations to a certain extent, but the direction of the wind affected it little. That was mainly because the pollution sources of NO_X_ were not only the traffic exhaust, but also the exhaust of the industrial factories nearby and thus NO_X_ was produced all around. With respect to NO_X_, the change of the CO concentration was affected by the wind direction. The monitoring van was at the west of the exhausting wind towers. Thus when the winds blew southwest or northwest, the van was located upwind. Thus, the monitoring results were influenced little by pollution from the tunnel, but mainly reflected the traffic pollution on the surface road. Some researchers have reported the similar result, which considered that the impact of pollution sources are much more significant than the effect of the wind. Chen [15] preliminary analyzed the influences of vehicular pollution sources and meteorological conditions for PM_10_ concentrations in the downtown streets in Beijing. The variation of concentrations in the streets (near the vehicular pollution sources) was affected by pollution sources greatly, and only at the conditions of unstable weather, the impact of pollution sources was little. Yang [16] calculated the contribution rates of the meteorological conditions and traffic control measurements for particle concentrations decreasing during the Beijing Olympics, the results showed that the traffic control measurements had a more apparent impact on decreasing the roadside PM_2.5_ concentrations.

### PM_2.5_ and the OC, EC content inside the tunnel

**Figure 8:**
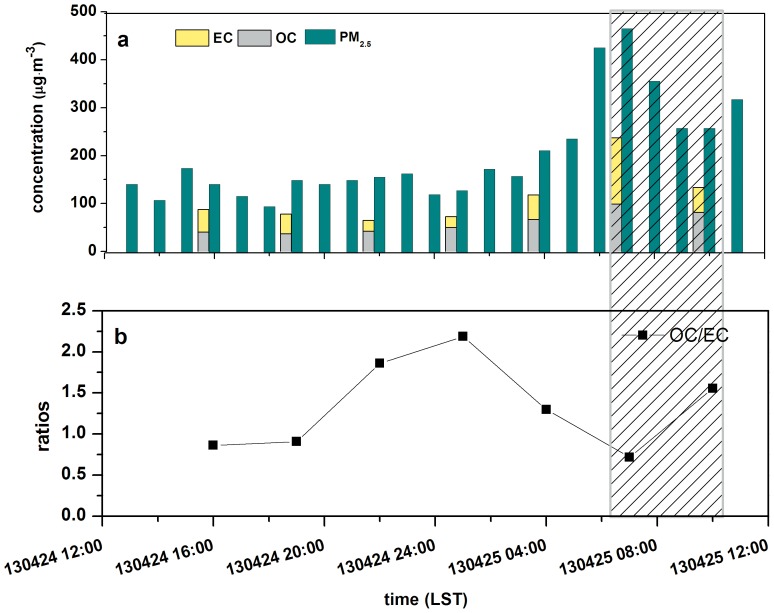
PM_2.5_ concentrations and OC and EC concentrations in PM_2.5_, as well as the OC/EC ratio inside the tunnel (shaded area: the period of the fan working).

The variation of PM_2.5_ concentrations inside the tunnel was shown in Fig. 8. During the morning rush hours, the concentrations climbed to the peak value of 468.45 µg/m^3^ sharply. Fig. 8 also gives the contents of organic carbon and elemental carbon in PM_2.5_ inside the tunnel as well as their ratios. OC concentrations varied from 37.09 µg/m^3^ to 99.06 µg/m^3^ while EC concentrations ranged from 22.69 µg/m^3^ to 137.99 µg/m^3^. Additionally, the contribution of OC and EC to PM_2.5_ were 21.15%–38.45% and 14.33%–32.83%, respectively. Thus, TC contributed to PM_2.5_ from 40.99% to 61.11%. From Fig. 8.a, it shows that high values of OC and EC appeared at the beginning of the morning rush hours, while low values of OC appeared at nightfall and low values of EC appeared at midnight. It could be noticed that the maximum of OC/EC ratio was 2.19, while the minimum was 0.72, with the mean value of 1.34. Comparing with other tunnel studies around the world, the mean OC/EC ratios were indicated in Table 1 [9], [17]–[25]. It seems that EC concentrations were higher than OC in the tunnel in many cases, and the ratios between OC and EC were less than 2 even in the case that OC concentrations were higher than EC. In general, the OC/EC ratios are more than 2 in the urban ambient air, as OC can be produced by photochemical reactions; while inside the tunnel under the circumstance of no light, it was difficult to generate secondary chemical reactions to produce more OC, so the OC/EC ratios were relatively smaller. On the other hand, EC was primarily emitted by HDVs, and thus fewer HDVs, the OC/EC ratios are larger instead. In addition, compared with the results of tunnel experiments in Guangzhou [9] and Shenzhen [23], it could be found that the mean of OC/EC in this study is higher than that of those two tunnel experiments. Heavy duty vehicles were accounting for 18.4% to 35.1% of the total traffic volume passing through the Zhujiang tunnel in Guangzhou with the averaged OC/EC of 0.42. And for the Tanglang Hill tunnel in Shenzhen, the mean OC/EC of light duty vehicles was 1.2 while that of heavy duty vehicles was 0.42. Under the condition of the 33.8% proportion of HDVs, the OC/EC ratio was 0.48. Then it could be inferred that the proportion of HDVs passed through the Xiangyin tunnel is relatively lower.

**Table 1:** Mean OC/EC ratios in PM_2.5_ of tunnel studies.

Tunnel	City	Mean OC/EC	Analytical method	Reference
Zhujiang tunnel	Guangzhou	0.49	Thermal optical transmittance	He et al. [9]
Tanglang Hill tunnel	Shenzhen	0.52	Thermal optical transmittance	Liu et al. [23]
Xueshan Tunnel	Taiwan	1.26	Thermal optical reflectance	Zhu et al. [25]
Mount Victoria tunnel	Wellington	1.00	Thermal optical reflectance	Ancelet et al. [17]
Janio Quadios tunnel	Sao Paulo	1.59	Thermal optical transmittance	Brito et al. [18]
Rodoanal Mario Covas tunnel	Sao Paulo	0.49	Thermal optical transmittance	Brito et al. [18]
Sepulveda tunnel	Los Angeles	0.76	Thermal optical reflectance	Gillies et al. [20]
Squirrel Hill tunnel	Pittsburgh	1.00	Thermal optical transmittance	Grieshop et al. [21]
Urban tunnel	Marseille	0.56	Thermal optical transmittance	Haddad et al. [19]
Kaisermühlen tunnel	Vienna	0.30	Thermal two step combustion	Handler et al. [22]
Marquês de Pombal tunnel	Lisbon	0.29∼0.37	Thermal optical transmittance	Pio et al. [24]
Xiangyin tunnel	Shanghai	1.34	Thermal optical reflectance	this study

## Conclusions

Through this study, it has been found out that the highest hourly average concentrations of CO, NO, NO_2_ and NO_X_ inside the tunnel were 13.223 mg/m^3^, 1.829 mg/m^3^, 0.291 mg/m^3^ and 3.029 mg/m^3^, respectively, while the lowest ones were 3.086 mg/m^3^, 0.344 mg/m^3^, 0.080 mg/m^3^ and 0.619 mg/m^3^. Concentrations of pollutants (CO, NO, NO_2_, NO_X_) were higher during the daytime (especially in the morning rush hours), and lower at night, which is related to the traffic condition in the tunnel. Additionally, the concentrations of pollutants (CO, NO, NO_2_ and NO_X_) inside the tunnel were higher than those outside the tunnel, about 7, 109, 2 and 18 times, respectively.

Referring to the impact of wind on the concentrations of the pollutants outside the tunnel, the effect of wind was not as significant as the impact of pollution sources in the case of slow wind.

PM_2.5_ concentrations climbed to the peak value of 468.45 µg/m^3^ during the morning rush hours. As for the contents of organic carbon and elemental carbon in PM_2.5_ inside the tunnel, those were 21.15%–38.45% and 14.33%–32.83%, respectively. Moreover, the maximum of OC/EC ratio was about 2.19, and the minimum was 0.72, while the mean value was 1.34. The ratios suggested that the proportion of HDVs through the Xiangyin tunnel might be relatively lower.

## Supporting Information

Table S1 **The correlation coefficient r boundary value table.**
(DOCX)
