# Supplementary material for: Study on the Traffic Air Pollution inside and outside a Road Tunnel in Shanghai, China
Source: PLoS One. 2014 Nov 11;9(11):e112195. doi: 10.1371/journal.pone.0112195 (PMC4227705; doi:10.1371/journal.pone.0112195)
Supplement: Table S1 — The correlation coefficient r boundary value table. (DOCX) [file pone.0112195.s001.docx]

Table S1 the correlation coefficient r boundary value table

| ν | P(2)  P(1) | 0.50  0.25 | 0.20  0.10 | 0.10  0.05 | 0.05  0.025 | 0.02  0.01 | 0.01  0.005 | 0.005  0.0025 | 0.002  0.001 | 0.001  0.0005 |
| --- | --- | --- | --- | --- | --- | --- | --- | --- | --- | --- |
| 1 |  | 0.707 | 0.951 | 0.988 | 0.997 | 1.000 | 1.000 | 1.000 | 1.000 | 1.000 |
| 2 |  | 0.500 | 0.800 | 0.900 | 0.950 | 0.980 | 0.990 | 0.995 | 0.998 | 0.999 |
| 3 |  | 0.404 | 0.687 | 0.805 | 0.878 | 0.934 | 0.959 | 0.974 | 0.986 | 0.991 |
| 4 |  | 0.347 | 0.608 | 0.729 | 0.811 | 0.882 | 0.917 | 0.942 | 0.963 | 0.974 |
| 5 |  | 0.309 | 0.551 | 0.669 | 0.755 | 0.833 | 0.875 | 0.906 | 0.935 | 0.951 |
| 6 |  | 0.281 | 0.507 | 0.621 | 0.707 | 0.789 | 0.834 | 0.870 | 0.905 | 0.925 |
| 7 |  | 0.260 | 0.472 | 0.582 | 0.666 | 0.750 | 0.798 | 0.836 | 0.875 | 0.898 |
| 8 |  | 0.242 | 0.443 | 0.549 | 0.632 | 0.715 | 0.765 | 0.805 | 0.847 | 0.872 |
| 9 |  | 0.228 | 0.419 | 0.521 | 0.602 | 0.685 | 0.735 | 0.776 | 0.820 | 0.847 |
| 10 |  | 0.216 | 0.398 | 0.497 | 0.576 | 0.658 | 0.708 | 0.750 | 0.795 | 0.823 |
| 11 |  | 0.206 | 0.380 | 0.476 | 0.553 | 0.634 | 0.684 | 0.726 | 0.772 | 0.801 |
| 12 |  | 0.197 | 0.365 | 0.457 | 0.532 | 0.612 | 0.661 | 0.703 | 0.750 | 0.780 |
| 13 |  | 0.189 | 0.351 | 0.441 | 0.514 | 0.592 | 0.641 | 0.683 | 0.730 | 0.760 |
| 14 |  | 0.182 | 0.338 | 0.426 | 0.497 | 0.574 | 0.623 | 0.664 | 0.711 | 0.742 |
| 15 |  | 0.176 | 0.327 | 0.412 | 0.482 | 0.558 | 0.606 | 0.647 | 0.694 | 0.725 |
| 16 |  | 0.170 | 0.317 | 0.400 | 0.468 | 0.542 | 0.590 | 0.631 | 0.678 | 0.708 |
| 17 |  | 0.165 | 0.308 | 0.389 | 0.456 | 0.529 | 0.575 | 0.616 | 0.622 | 0.693 |
| 18 |  | 0.160 | 0.299 | 0.378 | 0.444 | 0.515 | 0.561 | 0.602 | 0.648 | 0.679 |
| 19 |  | 0.156 | 0.291 | 0.369 | 0.433 | 0.503 | 0.549 | 0.589 | 0.635 | 0.665 |
| 20 |  | 0.152 | 0.284 | 0.360 | 0.423 | 0.492 | 0.537 | 0.576 | 0.622 | 0.652 |
| 21 |  | 0.148 | 0.277 | 0.352 | 0.413 | 0.482 | 0.526 | 0.565 | 0.610 | 0.640 |
| 22 |  | 0.145 | 0.271 | 0.344 | 0.404 | 0.472 | 0.515 | 0.554 | 0.599 | 0.629 |
| 23 |  | 0.141 | 0.265 | 0.337 | 0.396 | 0.462 | 0.505 | 0.543 | 0.588 | 0.618 |
| 24 |  | 0.138 | 0.260 | 0.330 | 0.388 | 0.453 | 0.496 | 0.534 | 0.578 | 0.607 |
| 25 |  | 0.136 | 0.255 | 0.323 | 0.381 | 0.445 | 0.487 | 0.524 | 0.568 | 0.597 |
| 26 |  | 0.133 | 0.250 | 0.317 | 0.374 | 0.437 | 0.479 | 0.515 | 0.559 | 0.588 |
| 27 |  | 0.131 | 0.245 | 0.311 | 0.367 | 0.430 | 0.471 | 0.507 | 0.550 | 0.579 |
| 28 |  | 0.128 | 0.241 | 0.306 | 0.361 | 0.423 | 0.463 | 0.499 | 0.541 | 0.570 |
| 29 |  | 0.126 | 0.237 | 0.301 | 0.355 | 0.416 | 0.456 | 0.491 | 0.533 | 0.562 |
| 30 |  | 0.124 | 0.233 | 0.296 | 0.349 | 0.409 | 0.449 | 0.484 | 0.526 | 0.554 |
| 31 |  | 0.122 | 0.229 | 0.291 | 0.344 | 0.403 | 0.442 | 0.477 | 0.518 | 0.546 |
| 32 |  | 0.120 | 0.226 | 0.287 | 0.339 | 0.397 | 0.436 | 0.470 | 0.511 | 0.539 |
| 33 |  | 0.118 | 0.222 | 0.283 | 0.334 | 0.392 | 0.430 | 0.464 | 0.504 | 0.532 |
| 34 |  | 0.116 | 0.219 | 0.279 | 0.329 | 0.386 | 0.424 | 0.458 | 0.498 | 0.525 |
| 35 |  | 0.115 | 0.216 | 0.275 | 0.325 | 0.381 | 0.418 | 0.452 | 0.492 | 0.519 |
| 36 |  | 0.113 | 0.213 | 0.271 | 0.320 | 0.376 | 0.413 | 0.446 | 0.486 | 0.513 |
| 37 |  | 0.111 | 0.210 | 0.267 | 0.316 | 0.371 | 0.408 | 0.441 | 0.480 | 0.507 |
| 38 |  | 0.110 | 0.207 | 0.264 | 0.312 | 0.367 | 0.403 | 0.435 | 0.474 | 0.501 |
| 39 |  | 0.108 | 0.204 | 0.261 | 0.308 | 0.362 | 0.398 | 0.430 | 0.469 | 0.495 |
| 40 |  | 0.107 | 0.202 | 0.257 | 0.304 | 0.358 | 0.393 | 0.425 | 0.463 | 0.490 |
| 41 |  | 0.106 | 0.199 | 0.254 | 0.301 | 0.354 | 0.389 | 0.420 | 0.458 | 0.484 |
| 42 |  | 0.104 | 0.197 | 0.251 | 0.297 | 0.350 | 0.384 | 0.416 | 0.453 | 0.479 |
| 43 |  | 0.103 | 0.195 | 0.248 | 0.294 | 0.346 | 0.380 | 0.411 | 0.449 | 0.474 |
| 44 |  | 0.102 | 0.192 | 0.246 | 0.291 | 0.342 | 0.376 | 0.407 | 0.444 | 0.469 |
| 45 |  | 0.101 | 0.190 | 0.243 | 0.288 | 0.338 | 0.372 | 0.403 | 0.439 | 0.465 |
| 46 |  | 0.100 | 0.188 | 0.240 | 0.285 | 0.335 | 0.368 | 0.399 | 0.435 | 0.460 |
| 47 |  | 0.099 | 0.186 | 0.238 | 0.282 | 0.331 | 0.365 | 0.395 | 0.431 | 0.456 |
| 48 |  | 0.098 | 0.184 | 0.235 | 0.270 | 0.328 | 0.361 | 0.391 | 0.427 | 0.451 |
| 49 |  | 0.097 | 0.182 | 0.233 | 0.276 | 0.325 | 0.358 | 0.387 | 0.423 | 0.447 |
| 50 |  | 0.096 | 0.181 | 0.231 | 0.273 | 0.322 | 0.354 | 0.384 | 0.419 | 0.443 |
| 60 |  | 0.087 | 0.165 | 0.211 | 0.250 | 0.295 | 0.325 | 0.352 | 0.385 | 0.408 |
| 70 |  | 0.081 | 0.153 | 0.195 | 0.232 | 0.274 | 0.302 | 0.327 | 0.358 | 0.380 |
| 80 |  | 0.076 | 0.143 | 0.183 | 0.217 | 0.257 | 0.283 | 0.307 | 0.336 | 0.357 |
| 90 |  | 0.071 | 0.135 | 0.173 | 0.205 | 0.242 | 0.267 | 0.290 | 0.318 | 0.338 |
| 100 |  | 0.068 | 0.128 | 0.164 | 0.195 | 0.230 | 0.254 | 0.276 | 0.308 | 0.321 |
| 150 |  | 0.055 | 0.105 | 0.134 | 0.159 | 0.189 | 0.208 | 0.227 | 0.249 | 0.254 |
| 200 |  | 0.048 | 0.091 | 0.116 | 0.138 | 0.164 | 0.181 | 0.197 | 0.216 | 0.230 |
| 250 |  | 0.043 | 0.081 | 0.104 | 0.124 | 0.146 | 0.162 | 0.176 | 0.194 | 0.206 |
| 300 |  | 0.039 | 0.074 | 0.095 | 0.113 | 0.134 | 0.148 | 0.161 | 0.177 | 0.188 |
| 350 |  | 0.036 | 0.068 | 0.088 | 0.105 | 0.124 | 0.137 | 0.149 | 0.164 | 0.175 |
| 400 |  | 0.034 | 0.064 | 0.082 | 0.098 | 0.116 | 0.128 | 0.140 | 0.154 | 0.164 |
| 450 |  | 0.032 | 0.060 | 0.077 | 0.092 | 0.109 | 0.121 | 0.132 | 0.145 | 0.154 |
| 500 |  | 0.030 | 0.057 | 0.074 | 0.088 | 0.104 | 0.115 | 0.125 | 0.138 | 0.146 |
| 600 |  | 0.028 | 0.052 | 0.067 | 0.080 | 0.095 | 0.105 | 0.114 | 0.126 | 0.134 |
| 700 |  | 0.026 | 0.048 | 0.062 | 0.074 | 0.088 | 0.097 | 0.106 | 0.116 | 0.124 |
| 800 |  | 0.024 | 0.045 | 0.058 | 0.069 | 0.082 | 0.091 | 0.099 | 0.109 | 0.116 |
| 900 |  | 0.022 | 0.043 | 0.055 | 0.065 | 0.077 | 0.086 | 0.093 | 0.103 | 0.109 |
| 1000 |  | 0.021 | 0.041 | 0.052 | 0.062 | 0.073 | 0.081 | 0.089 | 0.098 | 0.104 |
